# Supplementary material for: Histone Abundance Quantification via Flow Cytometry of Htb2-GFP Allows Easy Monitoring of Cell Cycle Perturbations in Living Yeast Cells, Comparable to Standard DNA Staining
Source: J Fungi (Basel). 2023 Oct 20;9(10):1033. doi: 10.3390/jof9101033 (PMC10608138; doi:10.3390/jof9101033)
Supplement: Supplementary file 1 [file jof-09-01033-s001.zip › jof-2568063-supplementary.pdf]

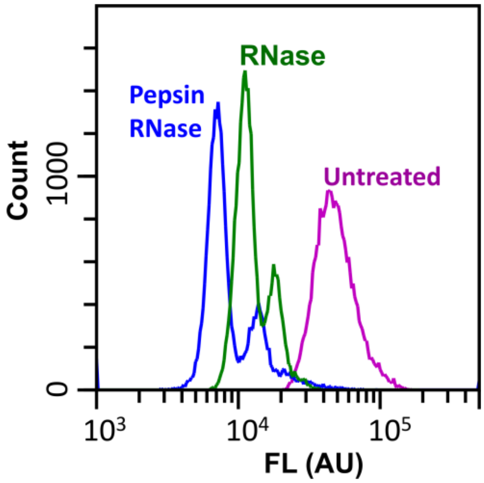

Figure S1. Pepsin treatment is not required either for efficient RNase treatment nor for PI staining. The *O. polymorpha* exponentially grown cells we subjected to the fixation-PI staining procedure described in Materials and Methods, which includes RNase treatment (RNase), or the procedure omitting the RNase treatment step (No treatment), or the procedure with additional pepsin treatment step prior to the RNase treatment (Pepsin RNase).

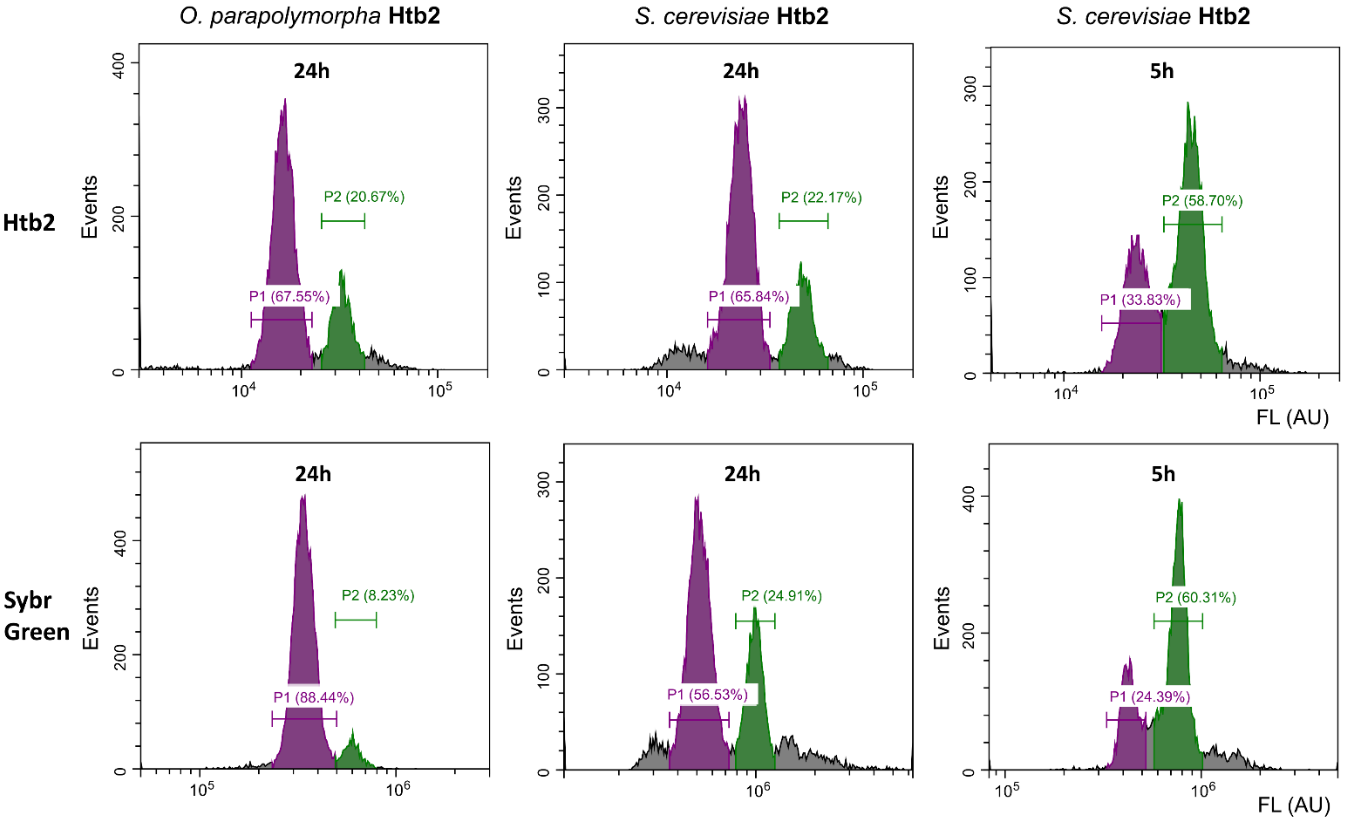

Figure S2. Gates for measuring the 1c and 2c

Map and sequence of the pKAM984 plasmid, scheme of its genome integration and positions of primers 610L1 (GACGGCTAAAGCGAGTGG), SL3 (GGTGATGCTGTCGCCGAAGAAG), AU (GGTGGAAATGAGCAGAGAGG), and AL (GGATCGTGAACAGCTTAGTGG), used for the PCR analysis of the integration locus.

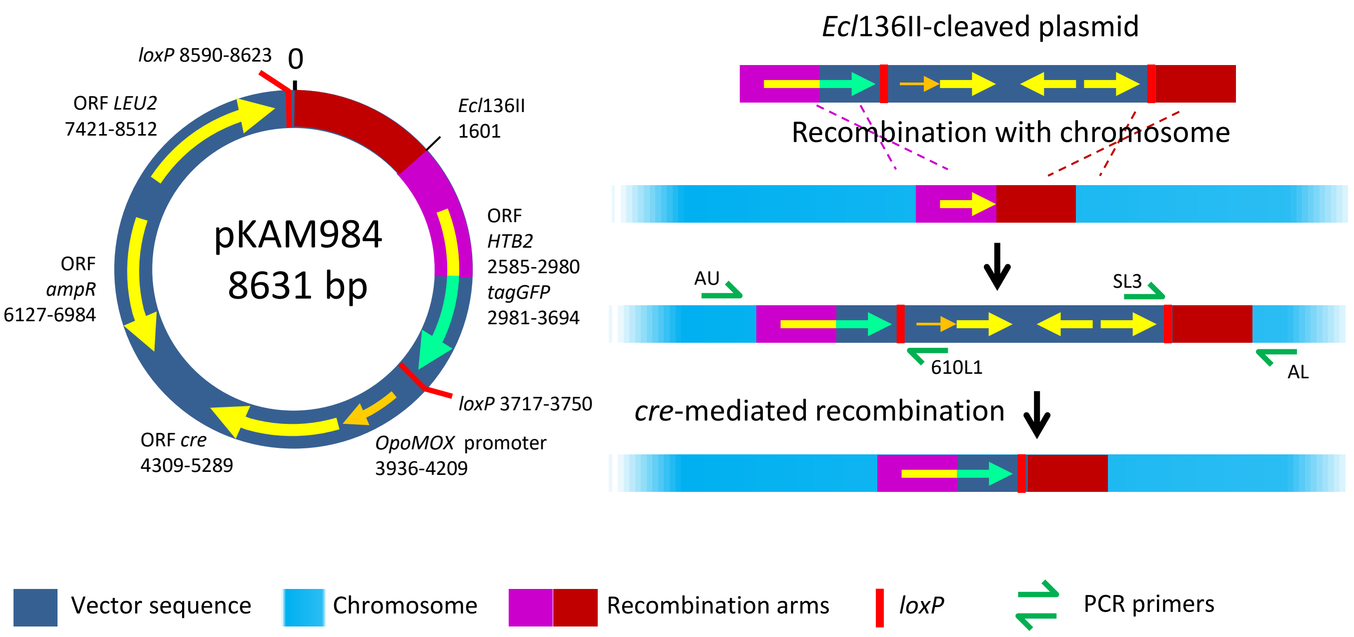

acgaagttattaggtgatggtacggtgtattgggtataattgtatattaggaatgagatttcagatcgaattagcgtaactgtcgtattccagtactggtactatgttgttcgatgcgctgtgcggcctcatcggcttgacacaacaacgtccctgtcgcaaagtaatattaggagataatattagta
gataatatttattataattatttgaaatataaaatcagtttgagctagaagcatgagatgtaaaatataattattagttacagttattatttttaccacattatactccttgaaatgcttctttattcttccacaaagctatcttgctccactgctgggcccttcccttttcctcgccctctgtaacaccgttt
cagctactatacaacaacttccatgtcatatcaaaagcattacgtcagcagctcactgccccatcgtaggctaaattaaatgtgcagaaagtcaagccttcagctgtttctttggccgatatttgcagcatggagataatcattcctacggttgacaatccttccagaacctcgaaggcggccctgtg
tggcatgtttctggcaaaatttgatgtgcatacgggatatgaattaaagtggttccgatcactaaatgagtctttatatcctagcaaaggcttggaattcaaggcattgccgtcggggctccactcagttgatactgacattatctgttttgttcacgaaaaaggagagccgaacctggccaacacc
aagtctctattgtatggaatacttgttttcaagcagaacttatcatcgcagaagacagttgatggaaacgttgaccgaaactctgtgagaatgtactcacttggggttctggttgacccggaacacttgaacgaaacatggatgcccagagtatattcatcttgctggaactacagaaatgaatta
aacaggcttgtcacaggatttctggattccggagaggaacaagagaagtacaacagtctcgaacaattttggacagtccacagattcaaaggacctaatgacttgagcactaaattcaaagatatcataaaaaacgtcaatataaatagaagaaagtctatccaaactcttaccagtgacgag
ccacagaatgcagatagagggactagctctgagagtggcatgacctcacgcaccttagcagacaccttcacagatgggaccttgccgaaaatggcagcgaattggatcaatacagtattgatgacgagcacatggtcaactatcttcttcccttgatggagatcctcggccctttggtataccg
attgtggaagctggcattgactcgtaaaaaaatcattatttatgcaccgcaaaactctcatcgctcgatcagcgagctgtccaagatcatctactgcgtttcgctgatatcgtcgattccaaaggatttgaagctcatgttgattaaatccagggtgatccagcaggaaaccttggaacatctcaca
ccgctatacaacgtgtgtgttaacgatatcgggtttttgaagtcacaaaagaatgggttcatagcctcaacttctgaccagatcattttggaaaagtcagagctcatgtggtcggagccacagctagaaccgatctaccaggctacacgtggatacatggaaatcaaccaacgggtggctctcttg
aaccagcgtctggaagtgattagcgatctgttgcagatgctcaaagaacagctgggacactcaaacgacgagtacctggaatttatcgtgattatgcttattggtgcagaggtgattgtgtcgatcatcaacatcattgtcgatatagtgggacaaagataatggagatgtatactacgtttgatc
tgtgaatttcttaccgaacactaatatacacttggaccctacctaatcttacaactcctgggaacccttggcctttggcagcttctttggaagcaactcggagttgatgtttggcaagacaccaccctgggcaatggtgacagagcccaaaagcttgttcaattcctcatcgtttctgatggccagca
acaagtgtcttgggacaattctggtcttcttgttgtctctggcagcgttaccagccaactccaagatttcggcagccaagtactccaagacggcagtcaagtagactggagcaccggaaccaactctctgagcgtagttaccctttctgagcaatctgtggattctaccaactgggaaggtcaaacc
agccttggaagatctggagaccgaagccttatctgcggaacctgctttaccacctttaccaccggacattttagttaatttgaaaagatgagacaggatggggtattatcttgtatttatgttgggatgcgcgctttggcaaattagttccatgatttactcccgcgtcgtgcgtaaaacgagggat
gccaaatttgataggcctaatttttactcgcggtggagcgtaaattttgtcccaacgcggtccgcttcatggaaaaacaatcacctacaactatataataacatcccagtcaccccaattgccctcctagatattaccttatttttaaaaaatggctccaaaagcagaaaagaaaccagcctccaag
gccccagccgaaaagaagccagccggaaagaaaaccgctgcctcctccgatgctaagaagagaaccaaggccagaaaggagacctactcctcctacatctacaaggttttgaagcagacccacccagacactggtatttcccagaaggctatgtccatcatgaactcattcgtgaacgacatc
ttcgagagaatcgcttccgaggcttccaagcttgctgcttacaacaaaaagtccactatttccgccagagagatccagactgccgtgagactgatcttgcctggagaattggccaagcacgctgtttctgagggtaccagagctgtcactaagtacacttctgccacctctgcttacatgtctggag
gtgaggagctgttcgctggaatcgttccagtgctgatcgagctggatggagacgtgcatggacacaagttctctgtgagaggtgaaggagaaggtgatgcagactacggaaagctggagatcaagttcatctgcaccactggcaaacttccagttccttggcctactcttgttaccactctgtgct
atggcatccagtgcttcgcaagatacccagagcacatgaagatgaacgacttcttcaagtctgctatgccagaaggctacatccaggagagaaccatccagttccaggacgatggcaagtacaagaccagaggtgaagtgaagttcgagggtgacactctggtgaacagaatcgagctgaa
aggcaaggacttcaaggaggatggcaacatccttggacacaagctggagtattcgttcaactctcacaacgtctacatcagaccagacaaagccaacaacggactggaagccaacttcaagaccagacacaacatcgagggtggaggtgttcagcttgctgaccactaccagaccaacgtgc
cacttggagatggaccagtcctgattccaatcaaccactacctgtccactcagaccaagatttccaaggacagaaacgaagccagagaccacatggtccttctggagtcgttctctgcctgctgtcacacgcatggaatggacgagctgtacagataagatctcgacaacccttaatataacttc
gtataatgtatgctatacgaagttattaggtctagaaaacccactcgctttagccgtcgcgtaaaacttgtgaatctggcaactgaggggttctgcagccgcaaccgaacttgtcgctccgaggacgcggctggatggtggcatgtgaggctctgtttgctggcgtagcctacaacgtgacctta
cctaaccggacggcgctacccactgctgtctgtgcctgctaccagaaaatcaccagagcagcagagggccgatgtggcaactggtggggtgtcggacaggctgtttctccacagtgcaaatgcgggtgaaccggccagaaagtaaattcttatgctaccgtgcagcgactccgacatccccagt
ttttgccctacttgatcacagatggggtcagcgctgccgctaagtgtacccaaccgtccccacacggtccatctataaatactgctgccagtgcacggtggtgacatcaatccaaagttactacaagacaaatagacaagtcaagttagataaaatggtaagttgccaatgccagaggcagcatg
tgcaagtgttctaacctttctagtcaatcgatgcaacgagtgatgaggttcgcaagaacctggcagatgcgttcagggatcgccaggcgttttctgagcatacctggaaaatgcttctgtccgtttgccggtcgtgggcggcatggtgcaagttgaataaccgggaatggtttcccgcagaacctg
aagatgttcgcggttatcttctatatcttcaggcgcgcggtctggcagtaaaaactatccagcaacatttgggccagctaaacatgcttcatcgtcggtccgggctgccacgaccaagtgacagcaatgctgtttcactggttatgcggcggatcagaaaagaaaacgttgatgccggtgaacgtg
caaaacaggctctagcgttcgaacgcactgatttcgaccaggttcgttcactcatggaaaatagcgatcgctgccaggatatacgtaatctggcatttctggggattgcttataacaccctgttacgtatagccgaaattgccaggatcagggttaaagacatctcacgtactgacggtgggagaa
tgttaatccatattggcagaacgaaaacgctggttagcaccgcaggtgtagagaaggcacttagcctgggggtaactaaactggtcgagcgatggatttccgtctctggtgtagctgatgatccgaataactacctgttttgccgggtcagaaaaaatggtgttgccgcgccatctgccaccagc
cagctatcaactcgcgccctggaagggatttttgaagcaactcatcgattgatttacggcgctaaggatgactctggtcagagatacctggcctggtctggacacagtgcccgtgtcggagccgcgcgagatatggcccgcgctggagtttcaataccggagatcatgcaagctggtggctggac
caatgtaaatattgtcatgaactatatccgtaacctggatagtgaaacaggggcaatggtgcgcctgctggaagatggcgattagccattaacgcgccatgtgagcaaaaggccagcaaaaggccaggaaccgtaaaaaggccgcgttgctggcgtttttccataggctccgcccccctgacg
agcatcacaaaaatcgacgctcaagtcagaggtggcgaaacccgacaggactataaagataccaggcgtttccccctggaagctccctcgtgcgctctcctgttccgaccctgccgcttaccggatacctgtccgcctttctcccttcgggaagcgtggcgctttctcatagctcacgctgtaggta
tctcagttcggtgtaggtcgttcgctccaagctgggctgtgtgcacgaaccccccgttcagcccgaccgctgcgccttatccggtaactatcgtcttgagtccaacccggtaagacacgacttatcgccactggcagcagccactggtaacaggattagcagagcgaggtatgtaggcggtgctac
agagttcttgaagtggtggcctaactacggctacactagaaggacagtatttggtatctgcgctctgctgaagccagttaccttcggaaaaagagttggtagctcttgatccggcaaacaaaccaccgctggtagcggtggtttttttgtttgcaagcagcagattacgcgcagaaaaaaaggat
ctcaagaagatcctttgatcttttctacggggtctgacgctcagtggaacgaaaactcacgttaagggattttggtcatgagattatcaaaaaggatcttcacctagatccttttaaattaaaaatgaagttttaaatcaatctaaagtatatatgagtaaacttggtctgacagttaccaatgctta
atcagtgaggcacctatctcagcgatctgtctatttcgttcatccatagttgcctgactccccgtcgtgtagataactacgatacgggagggcttaccatctggccccagtgctgcaatgataccgcgagacccacgctcaccggctccagatttatcagcaataaaccagccagccggaagggcc
gagcgcagaagtggtcctgcaactttatccgcctccatccagtctattaattgttgccgggaagctagagtaagtagttcgccagttaatagtttgcgcaacgttgttgccattgctacaggcatcgtggtgtcacgctcgtcgtttggtatggcttcattcagctccggttcccaacgatcaaggcga
gttacatgatcccccatgttgtgcaaaaaagcggttagctccttcggtcctccgatcgttgtcagaagtaagttggccgcagtgttatcactcatggttatggcagcactgcataattctcttactgtcatgccatccgtaagatgcttttctgtgactggtgagtattcaaccaagtcattctgagaat
agtgtatgcggcgaccgagttgctcttgcccggcgtcaatacgggataataccgcgccacatagcagaactttaaaagtgctcatcattggaaaacgttcttcggggcgaaaactctcaaggatcttaccgctgttgagatccagttcgatgtaacccactcgtgcacccaactgatcttcagcat
cttttactttcaccagcgtttctgggtgagcaaaaacaggaaggcaaaatgccgcaaaaaagggaataagggcgacacggaaatgttgaatactcatactcttcctttttcaatattattgaagcatttatcagggttattgtctcatgagcggatacatatttgaatgtatttagaaaaataaaca
aataggggttccgcgcacatttccccgaaaagtgccacctgacgtctaagaaaccattattatcatgacattaacctataaaaataggcgtatcacgaggccctttcgtcttcaagaattaactgtgggaatactcaggtatcgtaagatgcaagagttcgaatctcttagcaaccattattttttc
ctcaacataacgagaacacacaggggcgctatcgcacagaatcaaattcgatgactggaaatttttttgttaatttcagaggtcgcctgacgcatatacctttttcaactgattacatttcagcaatatatatatatatttcaaggatataccattctaatgtctgcccctaagaagatcgtcgttttgc
caggtgaccacgttggtcaagaaatcacagccgaagccattaaggttcttaaagctatttctgatgttcgttccaatgtcaagttcgatttcgaaaatcatttaattggtggtgctgctatcgatgctacaggtgtcccacttccagatgaggcgctggaagcctccaagaaggttgatgccgttttg
ttaggtgctgtgggtggtcctaaatggggtacaggtagtgttagacctgaacaaggtttactaaaaatccgtaaagaacttcaattgtacgccaacttaagaccatgtaactttgcatccgactctcttttagacttatctccaatcaagccacaatttgctaaaggtactgacttcgttgttgtcaga
gaattagtgggaggtatttactttggtaagagaaaggaagacgatggtgatggtgtcgcttgggatagtgaacaatacaccgttccagaagtgcaaagaatcacaagaatggccgctttcatggccctacaacatgagccaccattgcctatttggtccttggataaagctaatgttttggcctct
tcaagattatggagaaaaactgtggaggaaaccatcaagaacgaatttcctacattgaaggttcaacatcaattgattgattctgccgccatgatcctagttaagaacccaacccacctaaatggtattatcatcaccagcaatatgtttggtgacatcatctccgatgaagcctccgttatcccag
gttccttgggtttgttgccatctgcgtccttggcctctttgccagacaagaacaccgcatttggtttgtacgaaccatgccacggttctgctccagatttgccaaagaataaggtcaaccctatcgccactatcttgtctgctgcaatgatgttgaaattgtcattgaacttgcctgaagaaggtaaggc
cattgaagatgcagttaaaaaggttttggatgcaggtatcagaactggtgatttaggtggttccaacagtaccaccgaagtcggtgatgctgtcgccgaagaagttaagaaaatccttgcttaaaaagattctctttttttatgatatttgtacgtacaccttgtacgcagcgtcgaagccggtcga
gaacccttaatataacttcgtataatgtatgctatacgaagttattaggtgat

Map and sequence of the pKAM930 plasmid.

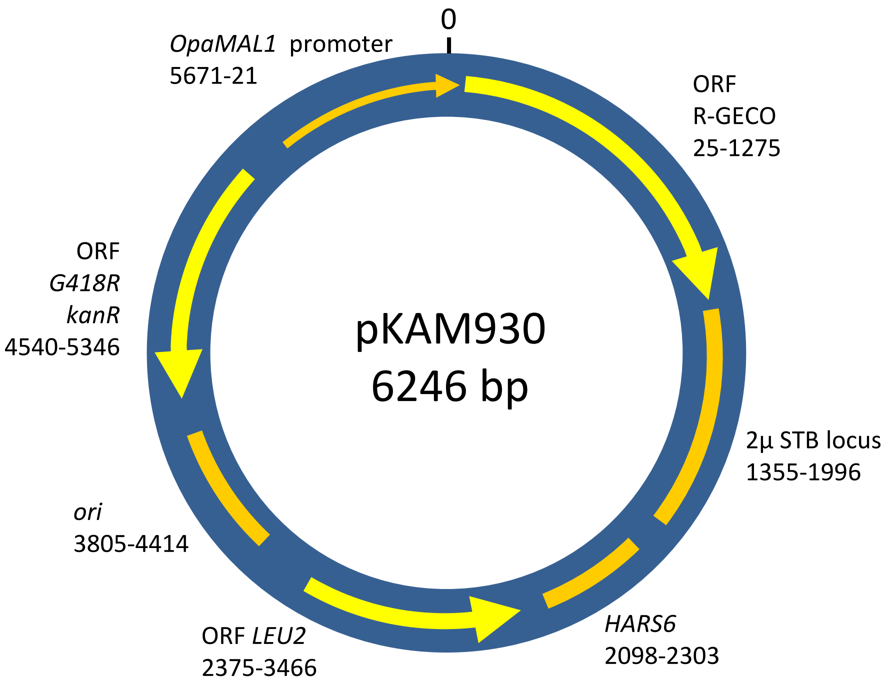

atccctcttgacaatattaattccatggtggactcgtcgagaagaaagtggaacaaggctggacacgctgtgagagccatcggaagactgtcgtctccagttgtgtctgagagaatgtacccagaggacggagcactgaagtc
ggagatcaagaagggactgagactgaaggacggaggtcactatgctgctgaggtgaagaccacctacaaagccaagaagccagtgcaactgccaggagcctacatcgttgacatcaagctggacatcgtgtctcacaacg
aggactacaccatcgttgagcagtgtgagagagccgaaggaagacactcgactggaggaatggacgaactctacaaaggtggaactggaggttctctggtgtccaaaggagaggaagacaacatggcaatcatcaagga
gttcatgcgcttcaaggttcacatggaaggctctgtgaacggacacgagttcgagatcgaaggtgagggtgaaggtagaccatacgaagccttccagactgccaagctgaaggtgaccaaaggtggaccacttccgtttgctt
gggacatcctgtctccacagttcatgtacggatcgaaagcctacatcaagcatccagcagacatcccagactacttcaagctgtcgtttccagaaggcttcagatgggagagagtgatgaacttcgaggatggaggcatcatc
catgtcaaccaagactcgtctctccaggatggagtgttcatctacaaggtgaagctgagaggaaccaacttccctccagatggaccagtgatgcagaagaagacgatgggatgggaagctaccagagaccaactcactgag
gaacagattgctgagttcaaggaagccttctcgctgttcgacaaggatggtgatggaaccatcactaccaaggagcttggaactgtgatgagatcgcttggacagaatccgactgaagcagaactccaggacatgatcaacg
aggttgatgctgatggagatggaaccttcgactttcctgagttcctgaccatgatggcaagaaagatgaacgacactgactctgaggaagagatcagagaagccttcagagtgttcgacaaggatggcaatggctacatcgg
agctgctgaactgagacacgttatgaccgaccttggtgagaagctgactgacgaagaggtggatgagatgatcagagttgctgacatcgacggagatggtcaggtgaactacgaggagttcgtgcagatgatgactgccaag
taatggatccgaattcgagctcggtacccggggatccaagcttcagctggcggccgcataggccactagtggatctgataacgaagcatctgtgcttcattttgtagaacaaaaatgcaacgcgagagcgctaatttttcaaac
aaagaatctgagctgcatttttacagaacagaaatgcaacgcgaaagcgctattttaccaacgaagaatctgtgcttcatttttgtaaaacaaaaatgcaacgcgagagcgctaatttttcaaacaaagaatctgagctgcatt
tttacagaacagaaatgcaacgcgagagcgctattttaccaacaaagaatctatacttcttttttgttctacaaaaatgcatcccgagagcgctatttttctaacaaagcatcttagattacttttttctccttttgtgcgctctataa
tgcagtctcttgataactttttgcactgtaggtccgttaaggttagaagaaggctactttggtgtctattttctcttccataaaaaaagcctgactccacttcccgcgtttactgattactagcgaagctgcgggtgcatttttcaag
ataaaggcatccccgattatattctataccgatgtggattgcgcatactttgtgaacagaaagtgatagcgttgatgattcttcattggtcagaaaattatgaacggtttcttctattttgtctctatatactacgataccgtcgatc
ttgcgattaacagtcgcatgccttaaaccagcttggccacgaagtcttatttggtgacatttctgctaggctgagaaccatatggatatcggaaaattcctgctattcgaaggtaatactagcagttgcattgagtcttgatattat
agaagggaattgtgctgattactaaatggccccggtcatatgttgtagccttttcagagccagaaatggttgctgcgaccttcaaaaaattaccacaattattaaaaaactcaaaaataaaaaatagccatcaaacagcgacc
ggtaactacaaaacctaagatcgacctcgagggggggcccggtacaaatatcataaaaaaagagaatctttttaagcaaggattttcttaacttcttcggcgacagcatcaccgacttcggtggtactgttggaaccacctaa
atcaccagttctgatacctgcatccaaaacctttttaactgcatcttcaatggccttaccttcttcaggcaagttcaatgacaatttcaacatcattgcagcagacaagatagtggcgatagggttgaccttattctttggcaaatct
ggagcagaaccgtggcatggttcgtacaaaccaaatgcggtgttcttgtctggcaaagaggccaaggacgcagatggcaacaaacccaaggaacctgggataacggaggcttcatcggagatgatgtcaccaaacatattg
ctggtgatgataataccatttaggtgggttgggttcttaactaggatcatggcggcagaatcaatcaattgatgttgaaccttcaatgtaggaaattcgttcttgatggtttcctccacagtttttctccataatcttgaagaggcca
aaacattagctttatccaaggaccaaataggcaatggtggctcatgttgtagggccatgaaagcggccattcttgtgattctttgcacttctggaacggtgtattgttcactatcccaagcgacaccatcaccatcgtcttcctttct
cttaccaaagtaaatacctcccactaattctctgacaacaacgaagtcagtacctttagcaaattgtggcttgattggagataagtctaaaagagagtcggatgcaaagttacatggtcttaagttggcgtacaattgaagttct
ttacggatttttagtaaaccttgttcaggtctaacactacctgtaccccatttaggaccacccacagcacctaacaaaacggcatcaaccttcttggaggcttccagcgcctcatctggaagtgggacacctgtagcatcgatag
cagcaccaccaattaaatgattttcgaaatcgaacttgacattggaacgaacatcagaaatagctttaagaaccttaatggcttcggctgtgatttcttgaccaacgtggtcacctggcaaaacgacgatcttcttaggggcag
acattagaatggtatatccttgaaatatatatatatattgctgaaatgtaatcagttgaaaaaggtatatgcgtcaggcgacctctgaaattaacaaaaaatttccagtcatcgaatttgattctgtgcgatagcgcccctgtgtg
ttctcgttatgttgaggaaaaaaataatggttgctaagagattcgaactcttgcatcttacgatacctgagtattcccacagttaattcttgaagacgaaagggcctcgtgatacgcctatttttataggttaatgtcatgtgagca
aaaggccagcaaaaggccaggaaccgtaaaaaggccgcgttgctggcgtttttccataggctccgcccccctgacgagcatcacaaaaatcgacgctcaagtcagaggtggcgaaacccgacaggactataaagatacca
ggcgtttccccctggaagctccctcgtgcgctctcctgttccgaccctgccgcttaccggatacctgtccgcctttctcccttcgggaagcgtggcgctttctcatagctcacgctgtaggtatctcagttcggtgtaggtcgttcgctc
caagctgggctgtgtgcacgaaccccccgttcagcccgaccgctgcgccttatccggtaactatcgtcttgagtccaacccggtaagacacgacttatcgccactggcagcagccactggtaacaggattagcagagcgaggt
atgtaggcggtgctacagagttcttgaagtggtggcctaactacggctacactagaagaacagtatttggtatctgcgctctgctgaagccagttaccttcggaaaaagagttggtagctcttgatccggcaaacaaaccaccg
ctggtagcggtggtttttttgtttgcaagcagcagattacgcgcagaaaaaaaggatctcaagaagatcctttgatcttttctacggggtctgacgctcagtggaacgaaaactcacgttaagggattttggtcatgagcttgcgc
cgtcccgtcaagtcagcgtaatgctctgccagtgttacaaccaattaaccaattctgattagaaaaactcatcgagcatcaaatgaaactgcaatttattcatatcaggattatcaataccatatttttgaaaaagccgtttctgt
aatgaaggagaaaactcaccgaggcagttccataggatggcaagatcctggtatcggtctgcgattccgactcgtccaacatcaatacaacctattaatttcccctcgtcaaaaataaggttatcaagtgagaaatcaccatg
agtgacgactgaatccggtgagaatggcaaaagtttatgcatttctttccagacttgttcaacaggccagccattacgctcgtcatcaaaatcactcgcatcaaccaaaccgttattcattcgtgattgcgcctgagcgagacga
aatacgcgatcgctgttaaaaggacaattacaaacaggaatcgaatgcaaccggcgcaggaacactgccagcgcatcaacaatgttctcaccagaatcaggatattcttctaatacctggaatgctgtttttccggggatcgc
agtggtgagtaaccatgcatcatcaggagtacggataaaatgcttgatggtcggaagaggcataaattccgtcagccagtttagtctgaccatctcatctgtaacatcattggcaacgctacctttgccatgtttcagaaacaac
tctggcgcatcgggcttcccatacaagcgatagattgtcgcacctgattgcccgacattatcgcgagcccatttatacccatataaatcagcatccatgttggaatttaatcgcggcctcgacgtttcccgttgaatatggctcata
acaccctatattatctttgtactaaagagcaattgataatgtgcgagaaaaactggtccttatataccgtttgcagcactccctcccgaactttacgaaaagtcgtgcgccacctgattttcatcacgccaaagacctacacgtat
gactactccgggccgctgtttcctgtgtgaaattgttatccgctcacaattccacacaacatacgagccggaagcataaagtgtaaagcctggggtgcctaatgagtgagctaactcacattaattgcgttgcgctcactgcccg
ctttccagtcgggaaacctgtcgtgccagatcttcgggagtatcgcaatagaaagcaggcactgtgcagattacgcgacatgatagctttgtatgttctacagactctgccgtaccactctacatataatgtgagagctctgcag
cgtcgtaatgaacacttgggttatacaagtttcctaagagccctttgacgttgattgctctggcttccctacgggctctcatgtggttcaagtgactccgcagtggctagcaagcgtgggggtcaattacgtcacttctattcatgta
ccccagactcaattgttggcagttatttcagcgagaattaaatgtgcaaattaattctcgctcaaataaattaggccgtgatttaatactcgctgaaacaggatcttgtctggggtacagataacaatcaaataactattatggac
gtgcataggaggtggagcccatgacgcaaacggaaatcctctttttattttcgggaagttaggaggataccaaagcgtcgcgctcgctaaagtgatgagaatgtctttagtaagctcaagccatataaagaccctccgcctcca
caattttttttatccctcttgacaat
